# Development of a high-performance and cost-effective in-vacuum undulator

**DOI:** 10.1107/S1600577524005873

**Published:** 2024-08-01

**Authors:** Kei Imamura, Yuichiro Kida, Akihiro Kagamihata, Takamitsu Seike, Shigeru Yamamoto, Haruhiko Ohashi, Takashi Tanaka

**Affiliations:** a Japan Synchrotron Radiation Research Institute Koto 1-1-1 Sayo Hyogo679-5198 Japan; b Photon Factory, Institute of Material Structure Science High Energy Accelerator Research Organization KEK, Oho 1-1 Tsukuba Ibaraki305-0801 Japan; c RIKEN SPring-8 Center, Koto 1-1-1, Sayo, Hyogo679-5148, Japan; University of Tokyo, Japan

**Keywords:** in-vacuum undulator

## Abstract

In-vacuum undulators (IVUs), which have become an essential tool in synchrotron radiation facilities, have two technical challenges toward further advancement: one is a strong attractive force between top and bottom magnetic arrays, and the other is a stringent requirement on magnetic materials to avoid demagnetization. The former imposes a complicated design on mechanical and vacuum structures, while the latter limits the possibility of using high-performance permanent magnets. To solve these issues, a number of technical developments have been made, such as force cancellation and modularization of magnetic arrays, and enhancement of resistance against demagnetization by means of a special magnetic circuit. The performance of a new IVU built upon these technologies has revealed their effectiveness for constructing high-performance IVUs in a cost-effective manner.

## Abstract 2

A high-performance in-vacuum undulator has been developed in a cost-effective manner.

## 1. Introduction

In-vacuum undulators (IVUs), in which magnetic arrays are placed inside a vacuum chamber and the achievable magnetic field can be significantly higher than that of out-vacuum undulators (OVUs), have nowadays become a mature and important tool in synchrotron radiation and X-ray free-electron laser (XFEL) facilities. The reduction of the minimum gap of even a few millimetres brings a great impact on the achievable performance, particularly for short-period undulators. After a first demonstration at KEK (Yamamoto *et al.*, 1992 ▸) and successful operation as standard insertion devices at SPring-8 (Hara *et al.*, 1998 ▸), IVUs have been widely adopted at many synchrotron radiation and XFEL facilities; it is reasonable to say that most of the undulators currently in operation with magnetic period shorter than 30 mm are of this type.

In spite of the advantages mentioned above, IVUs based on conventional design have several technical issues regarding the manufacturing cost and limitation on the achievable performance. In particular, the former may be a common issue in facilities that need a large number of IVUs, namely new facilities to be built from scratch or existing ones planning a major upgrade. For example, in the upcoming SPring-8-II project, or a major upgrade of SPring-8, more than 30 IVUs currently in operation should be replaced with shorter ones that can fit into the shorter straight section planned in the new storage ring. To proceed the SPring-8-II project within a limited budget, it is essential to establish a procedure to construct many IVUs in a cost-effective manner without sacrificing performance. For this purpose, we established a new IVU concept, referred to as ‘IVU-II’ (IVU for SPring-8-II). In this paper, we present the fundamental technologies developed for the IVU-II concept, and report its actual performance.

## 2. Technical issues in conventional IVUs

We first describe several technical issues with conventional IVUs preventing cost reduction and limiting achievable performance.

### 2.1. Structural difficulty

**Figure 1:**
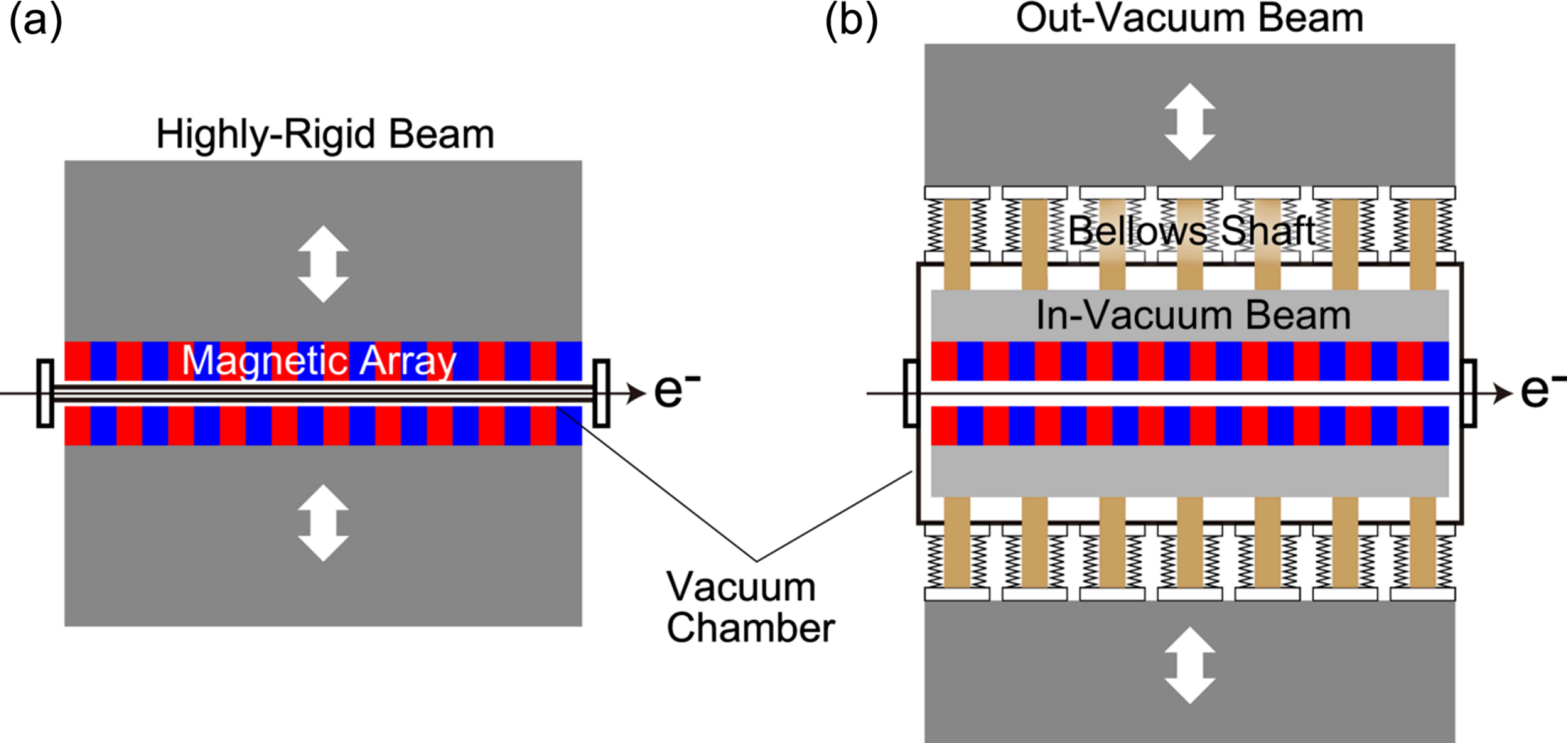
Overall structure of an undulator. (*a*) OVU. (*b*) IVU.

It is well known that a strong attractive force acts between the top and bottom magnetic arrays in an undulator, which exponentially depends on the gap in between and usually reaches a few tons in total. Thus, we need a mechanical frame of high rigidity that is capable of controlling the gap precisely under such a strong attractive force. Furthermore, we have another structural difficulty in conventional IVUs, as explained using Fig. 1 ▸.

Fig. 1 ▸(*a*) shows a schematic drawing of an OVU, where magnet blocks are directly mounted on a highly rigid beam to form a magnetic array; because the beam is sufficiently thick and is made of a material with high stiffness, deformation due to the strong attractive force is negligible and thus the local gap variation along the undulator axis is small enough to avoid any quality degradation. In IVUs, as illustrated in Fig. 1 ▸(*b*), we need another (in-vacuum) beam to place the magnetic array inside the vacuum chamber, whose stiffness is usually much lower than that of the out-vacuum beam. As a result, we need to connect the two beams with many shafts equipped with bellows to allow for the gap motion (bellows shaft), so that the deformation of the in-vacuum beam is within an acceptable level. It is obvious that such an increase of components imposes a complicated design, requires more assembly work, and brings a potential risk such as vacuum leakage; all of these put upward pressure on the cost of IVUs.

### 2.2. Magnetic material

In the selection of the permanent magnet (PM) materials for any applications, we need a compromise between two properties that are negatively correlated: remanent field (*B*_r_) and intrinsic coercivity (*H*_cj_). For application to undulators, *H*_cj_ usually takes priority, because PM blocks assembled to form an undulator magnetic circuit are demagnetized much more easily than those used alone, because of a strong reverse field acting on the surface facing the electron beam. Furthermore, PMs for IVUs should have higher *H*_cj_ than those for OVUs for two reasons. First, they are usually subject to higher radiation dose because of a much narrower operational gap. Second, they should undergo a bake-out process to achieve ultra-high-vacuum compatibility with the operation in a storage ring. Although the latter is not an issue in XFEL facilities driven by linear accelerators whose vacuum condition is much more relaxed, the former issue is so serious that we still need high *H*_cj_ to be used in IVUs. The requirement on *H*_cj_ eventually limits the availability of PM materials with high *B*_r_, putting a practical limit on the achievable performance of IVUs.

## 3. Key technologies

To overcome the technical challenges mentioned in the former sections and establish the IVU-II concept, we have developed several key technologies, the details of which are presented in the following sections.

### 3.1. Force cancellation

It is easy to understand that the structural difficulties in conventional IVUs mentioned above can be completely overcome if the attractive force acting on the magnetic array is cancelled out. In practice, this is not an easy task, because the attractive force *F*_a_ depends exponentially on the undulator gap *g*. Namely, we generally have a relation
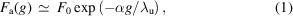
where λ_u_ is the undulator period, *F*_0_ is the maximum force and α is a parameter that depends on the specification of the magnetic array: if it has the so-called Halbach configuration, α is exactly 2π. In any case, the attractive force is given by an exponential decaying function of the gap, and thus it cannot be fully cancelled in a straightforward manner.

To date, several methods have been proposed and developed to reduce the mechanical load acting on the in-vacuum beam in IVUs, by means of springs (Marcouille *et al.*, 2010 ▸) and additional magnetic arrays (Bizen *et al.*, 2004 ▸). In the former method, many springs are placed on the side of the magnetic array to generate a repulsive force and reduce the mechanical load. The point is that the springs are divided into two groups (longer and shorter ones) working in different gap ranges, enabling the reduction of the mechanical load by part. Although this method is rather simple and cost-effective, there are two difficulties in the IVU-II concept. First, the attractive force cannot be completely cancelled out because of the linear response of the springs. Second, the springs interfere with the magnetic measurement along the longitudinal direction based on a Hall probe sensor.

**Figure 2:**
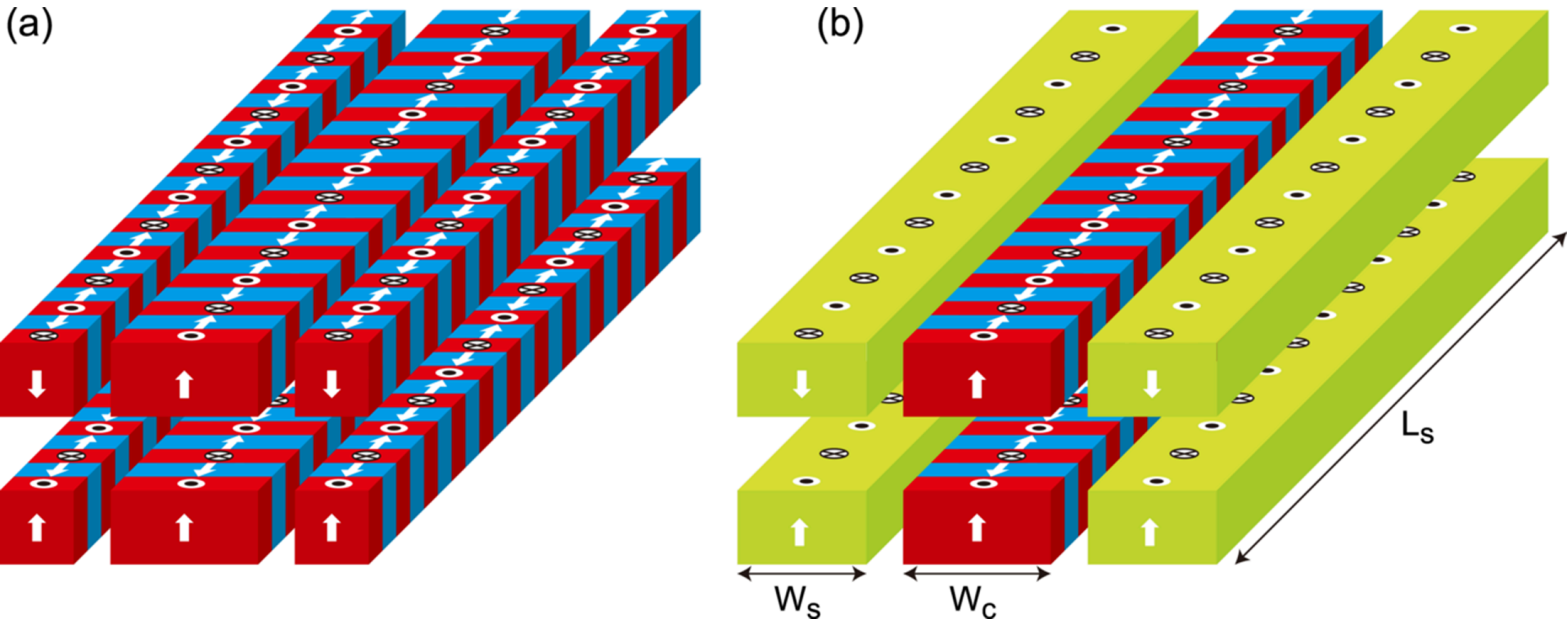
Schematic drawing of the magnetic configuration used to cancel out the attractive force by (*a*) additional magnetic arrays and (*b*) MMMs.

In the latter method, four additional magnetic arrays with period λ_u_ are placed to the sides as shown in Fig. 2 ▸(*a*), in a phase to generate a repulsive force. Ideally, the attractive force can be fully cancelled out using this method, because the repulsive force generated by the additional side arrays depends on the gap in an identical way to the attractive force. Because the gap of the side array can be comparable with or larger than the central array, it does not interfere with the magnetic measurement. Although this method is ideal, we should pay attention to the cost increase coming from the larger number (three times) of PM blocks.

To take advantage of the latter (magnetic) method in a more cost-effective manner, we decided to make use of monolithic multipole magnets (MMMs) that were originally proposed to facilitate the assembly of magnetic arrays for extremely short-period undulators (Yamamoto, 2013 ▸). Attempts were pursued and brought successful results to fabricate the short-period undulator magnets based on this method which included a connection method of MMMs to form a longer magnet array (Yamamoto, 2018 ▸). A light source characterization experiment was performed successfully by using 4 mm-period MMMs within a 35 MeV S-band linac (Yamamoto *et al.*, 2019 ▸).

As schematically shown in Fig. 2 ▸(*b*), the side arrays are replaced with PM blocks (MMMs) that are much longer than λ_u_ but periodically magnetized in the vertical direction with period λ_u_. As a result, the increase of PM blocks can be significantly suppressed compared with the original method for force cancellation. The length of the MMM (*L*_s_), which is chosen from the technical (manufacturing) and practical (handling) points of view, is around 150 mm.

Note that the magnetic field strength generated by the MMMs is about 30% lower than that generated by the conventional Halbach (central) array. This comes from the difference in the number of magnet blocks (*M*) per period (Halbach, 1983 ▸); *M* is exactly 4 in the central array, while it is effectively 2 in the MMMs. Recalling that the magnetic force is proportional to the square of the field strength, the width of the MMM (*W*_s_) should be comparable with that of the central array (*W*_c_) so that the repulsive and attractive forces balance. Besides this geometrical condition, the gap of the side array can be adjusted to finely tune the repulsive force and cancel the attractive force. Note that the force cancellation can be done perfectly only at a specific gap, because of the difference in *M* between the central array (*M* = 4) and MMMs (*M* = 2); to be more specific, the magnetic field generated by MMMs has a third-harmonic content which is not usually found. It should be emphasized, however, that its contribution is much lower than the fundamental component and can be practically neglected as discussed later.

The force cancellation achieved with the above scheme obviously simplifies the design of the mechanical structure; for example, we can significantly reduce the number of supporting points of the in-vacuum beam (bellows shafts), giving the possibility to take away the out-vacuum beam from the conventional design. This not only simplifies the structure but also reduces the total weight of the mechanical frame.

### 3.2. Modularization of the magnetic array

In the conventional IVU, each PM block is assembled into a holder (keeper) to form a magnet unit, and each magnet unit is mounted on the in-vacuum beam to form a magnetic array. The properties of all the magnet units are often measured before assembling to optimize the initial arrangement (unit sorting). Assuming a Halbach array with period 24 mm and length 3 m as an example, we need to deal with 1000 PM blocks, meaning that the assembly and measurement of the magnet unit should be repeated 1000 times. If the sorting does not work well, which is often the case because of the large number of PM blocks, we need to rearrange the PM blocks based on the measurement of the magnetic array after assembly (Tanaka *et al.*, 2001 ▸). In addition, requirement on the flatness of the surface of the in-vacuum beam and mechanical tolerance of machining the magnet holders and PM blocks may be stringent to guarantee the uniformity of the gap along the longitudinal axis. Thus, the construction of a high-quality magnetic array is a time-consuming task that requires special skills and expertise.

To overcome the difficulties mentioned above, we introduce a modularized structure, in which the magnetic array is composed of a number of magnet modules. In each module, many PM blocks are assembled onto a common base to form a ‘sub-undulator’ of length ∼150 mm, thus corresponding to five to seven magnetic periods for λ_u_ of around 24 mm. This modularization brings great advantages over the conventional unit structure. First, the number of mechanical parts (holder) is considerably reduced. Second, the modular structure to assemble the PM blocks onto the handy-sized base instead of the long (more than a meter) in-vacuum beam allows for assembly assisted by precisely controlled actuators, or even gives the possibility of a robotic assembly as was first implemented in the manufacturing of IVUs for SwissFEL (Calvi *et al.*, 2018 ▸). Third, the requirement on the flatness of the in-vacuum beam can be relaxed by inserting a shim plate on the bottom of each module and adjusting its thickness if necessary, which is a common technique widely used in manufacturing and tuning of insertion devices. The module structure is also convenient to accommodate the MMMs. One concern regarding the modular structure is the possibility of virtual leak caused by localized dimples on the surface of the in-vacuum beam. We have to make sure that its surface is machined carefully to avoid this issue; machining grooves for evacuation is an alternative solution.

Besides the above points, another important advantage of modularization is that all magnet modules can be assessed before assembling. To be specific, the field integral of each module can be measured quite precisely by means of a stretched wire scheme with an improvement being made in which the target object (magnet module) is moved transversely with respect to the stretched wire. Because the wire is rigidly fixed and is free from any vibration that can potentially bring unwanted noise, the measurement error is reduced below 10^−3^ T mm, which is roughly one order of magnitude better than the conventional method with a moving wire. In addition, it can measure the net field integral of each module, without the ambient field.

The field integral data measured in the above process are then used to optimize the arrangement of each magnet module (module sorting) to reduce the integrated multipole and trajectory wander. Then a magnetic force acting on each pair (top and bottom) of modules is measured using a three-dimensional (3D) load cell to make sure that it is below an acceptable level before mounting on the in-vacuum beam.

### 3.3. Inclined Halbach configuration

**Figure 3:**
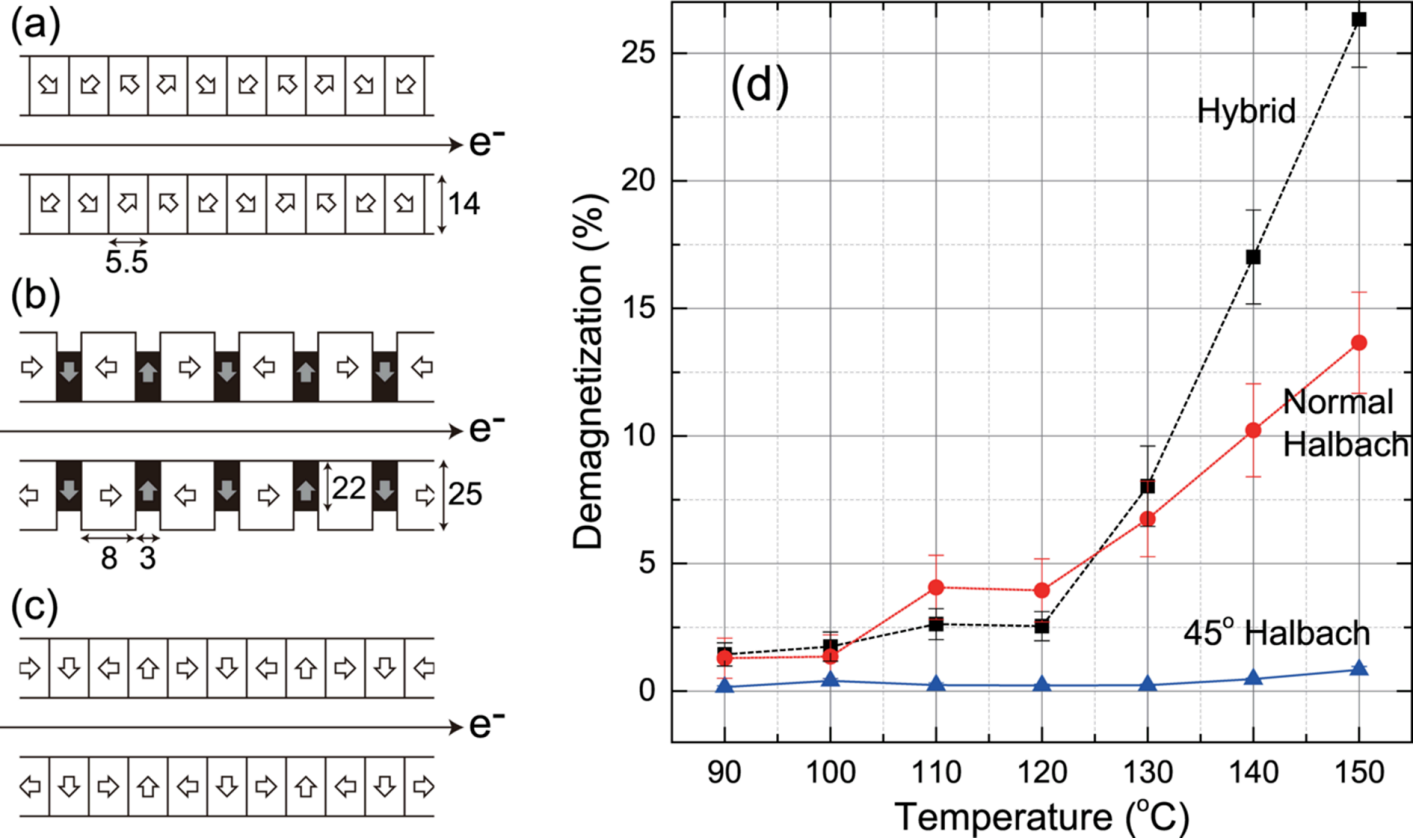
Schematic illustration of undulator magnetic circuits. (*a*) 45° inclined Halbach, (*b*) hybrid and (*c*) normal Halbach configurations, and (*d*) dependence of the thermal demagnetization on the magnet configuration. Note that the measurement error in the 45°-Halbach configuration is less than the symbol size.

Unlike the two key technologies to overcome the structural difficulties and reduce the manufacturing cost explained above, the third one leads to a practical enhancement of the performance. As mentioned in Section 2.2, the PM material for IVUs should have high *H*_cj_, and thus its *B*_r_ is relatively low. To expand the availability of PM materials for IVUs, we have proposed a new undulator magnetic circuit (45°-Halbach configuration) as shown in Fig. 3 ▸(*a*), which is much more resistive against demagnetization than conventional ones, *i.e.* hybrid [Fig. 3 ▸(*b*)] and normal Halbach [Fig. 3 ▸(*c*)] configurations (Bizen *et al.*, 2018 ▸; Tanaka & Kagamihata, 2021 ▸).

Fig. 3 ▸(*d*) shows the experimental results from examining the thermal demagnetization of three undulator samples assembled with the three different magnet configurations shown in (*a*)–(*c*), using the same PM material with *B*_r_ = 1.37 T, which is roughly 20% higher than that of conventional PM material for IVUs. All the samples have the same magnetic period of 22 mm, and the dimensions of each PM block (empty rectangle) and pole piece (solid rectangle) are indicated in Fig. 3 ▸. The width of each PM block is 30 mm for the Halbach configurations [Figs. 3 ▸(*a*) and 3 ▸(*c*)], and that for the hybrid configuration [Fig. 3 ▸(*b*)] is 35 mm with the width of the pole piece being 25 mm. The magnetic field 2.5 mm above the surface of each sample was measured before and after heating up to respective target temperatures, and the demagnetization rate is defined as the variation of the peak field normalized by that at room temperature.

Note that a grain boundary diffusion process (Nakamura *et al.*, 2005 ▸; Hirota *et al.*, 2006 ▸) has been applied to the PM blocks to improve *H*_cj_ from 1200 kA m^−1^ to 1850 kA m^−1^ without sacrificing *B*_r_, albeit only at the edge and surface of each PM block. Regardless of this improvement, the undulator samples with the conventional magnet circuits are demagnetized even below 100°C. In particular, the demagnetization in the hybrid configuration is more significant than in the normal Halbach configuration above 120°C; this comes from a strong reverse field localized at the corner of the PM blocks as discussed in a former paper (Bizen *et al.*, 2018 ▸). Recalling that the magnetic arrays for IVUs are heated up to a temperature higher than 100°C during the bake-out process, this PM material cannot be used for IVUs as long as the conventional configuration is supposed. In contrast, we do not find any demagnetization in the sample with the 45°-Halbach configuration at least below 140°C. Summarizing the above result, it is reasonable to say that the 45°-Halbach configuration effectively enhances the performance of IVU by ∼20%.

## 4. Construction and performance of IVU-II

Based on the technical developments presented in the former sections, we have built several IVU-IIs to replace the conventional IVUs currently in operation at SPring-8. As an example, we report the performance of an IVU-II with magnetic length of 3.3 m and period of 28 mm (hereafter referred to as IVU-II28) to discuss the effectiveness of the key technologies.

**Figure 4:**
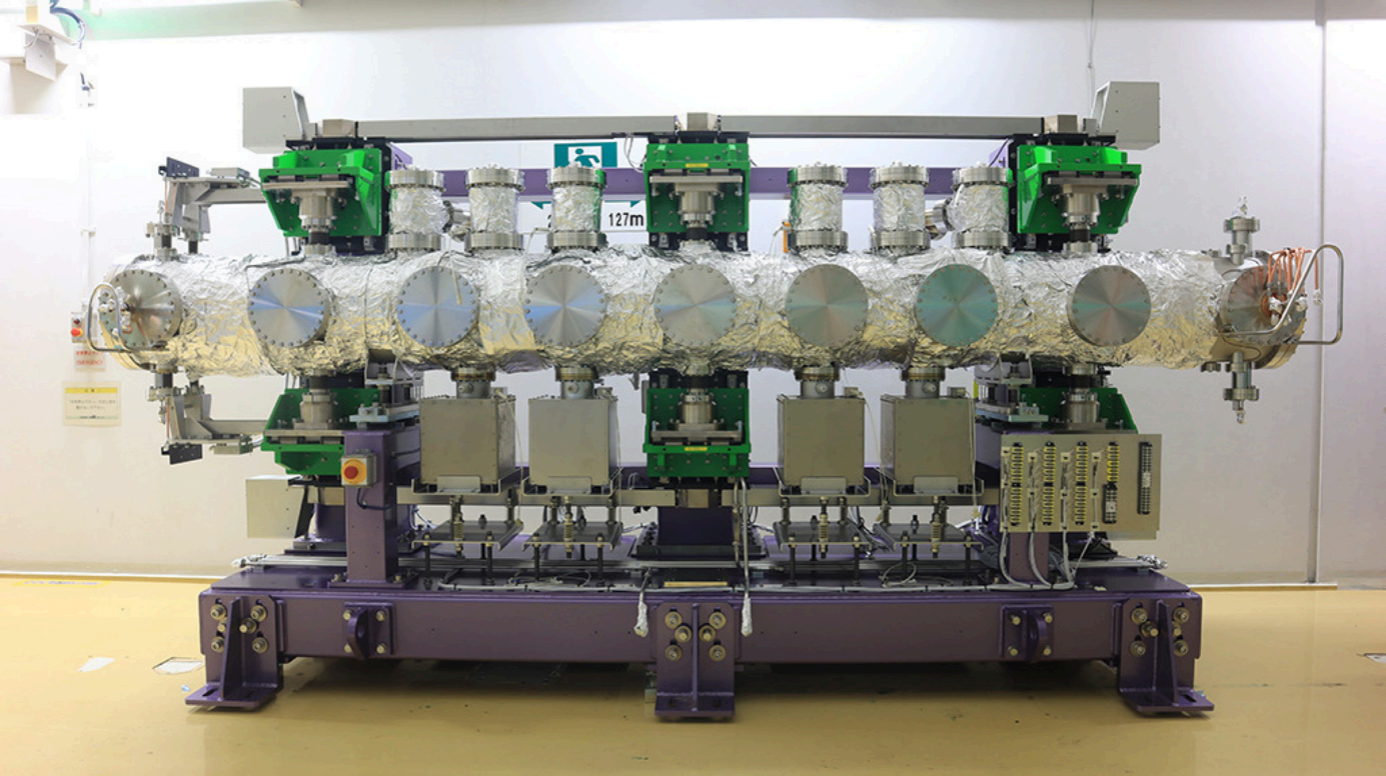
Photograph of the overall view of IVU-II28. Three bellows shafts support the in-vacuum beam without the out-vacuum beam.

Fig. 4 ▸ shows a photograph of the overall view of IVU-II28. Three bellows shafts support the in-vacuum beam, and are directly connected to the actuation system to open and close the gap, without the out-vacuum beam. In the top and bottom sides of the vacuum chamber that are usually occupied by the out-vacuum beam in the conventional IVUs, NEG (non-evaporated getter) and ion pumps are assembled.

**Figure 5:**
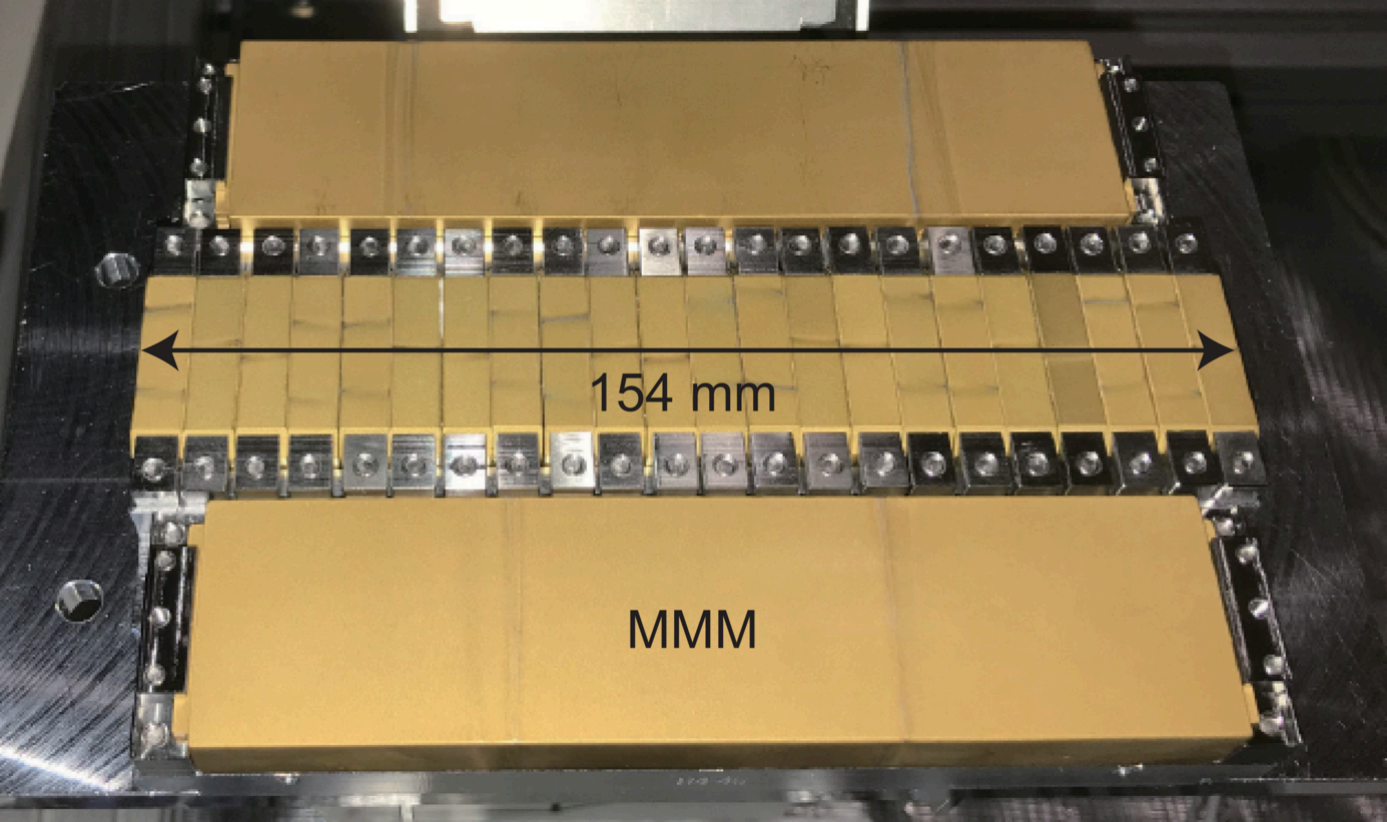
Photograph of a magnet module with magnetic period of 28 mm and total length of 154 mm corresponding to 5.5 periods.

Fig. 5 ▸ shows a photograph of a magnet module for IVU-II28, where 22 PM blocks are mounted on a common base to form a 5.5 period (154 mm-long) sub-undulator. After assembly, field integrals of respective modules were measured at a position 3 mm above the surface (corresponding to a gap of 6 mm), to optimize their locations and orientations (module sorting). Note that each MMM is slightly shorter than the module in order to give extra space, which is used to assemble the MMM on the base by mechanical clamps, and to insert a small magnet to correct the electron trajectory if necessary.

**Figure 6:**
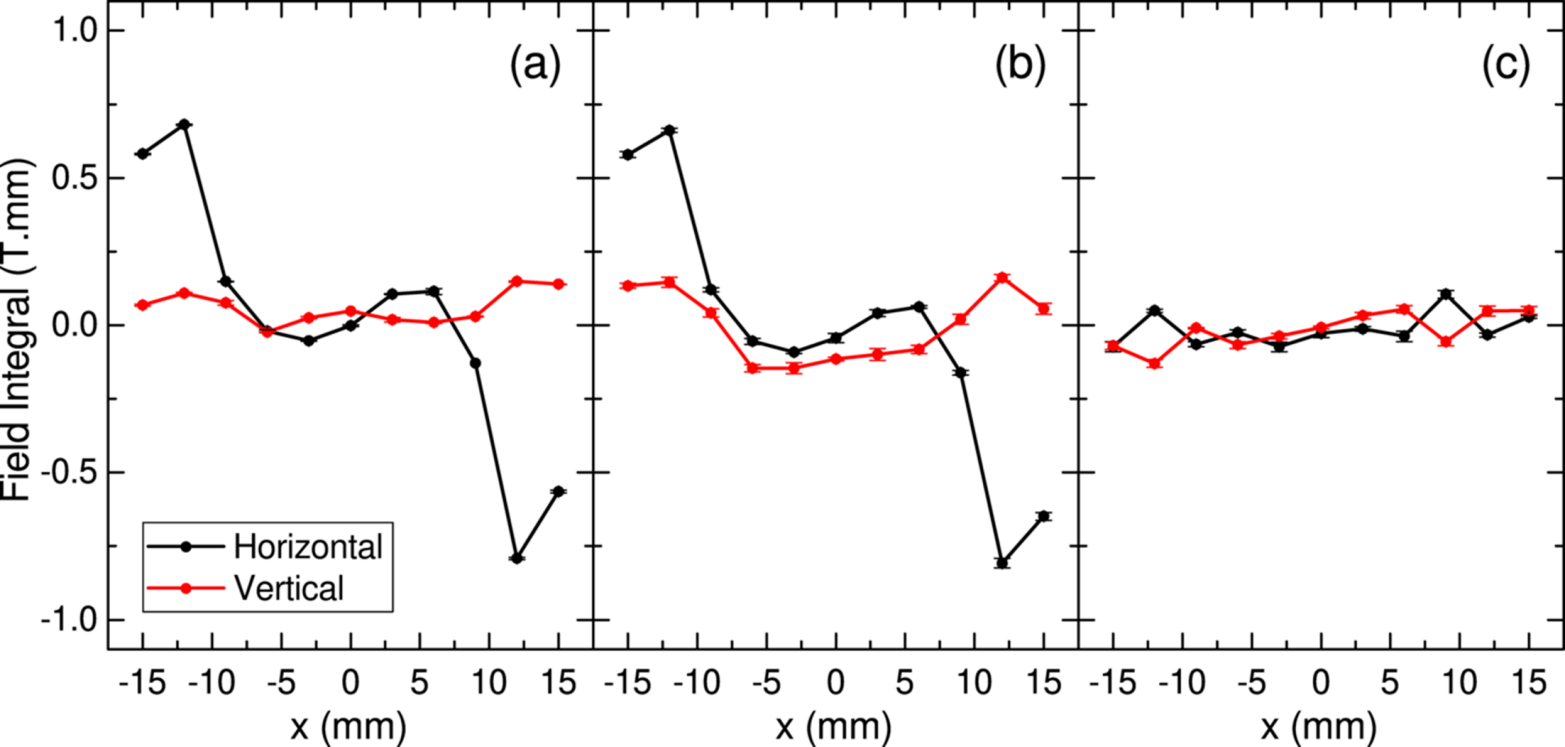
Field integrals of IVU-II28: (*a*) evaluated by summing up the measured results of respective magnet modules, (*b*) measured after assembling the modules to form a pair of magnetic arrays, and (*c*) after correction by the magic fingers.

Fig. 6 ▸(*a*) shows the horizontal and vertical field integrals predicted by the module sorting, given by summing up the measured field integrals with the optimized module arrangement taken into account. The results are consistent with the field integrals actually measured for the whole magnetic array after assembly, shown in Fig. 6 ▸(*b*); this suggests the validity of several processes for constructing the magnetic array such as measurement, sorting and assembling.

It should be noted that the measurement result shown in Fig. 6 ▸(*b*) may not be acceptable as a practical undulator, because it contains a large amount of integrated multipoles. In principle, this may be reduced by the unit sorting, in which the location and orientation of each PM block in a module are optimized by the field integral measurement of all the PM blocks. For this purpose, however, we have to repeat the measurement many times and deal with a huge number of PM blocks and data sets. We skip such a time-consuming process and just focus on the measurement of each module, to ‘moderately’ optimize the initial arrangement of PM blocks. In practice, an integrated multipole that is not too large as shown in Fig. 6 ▸(*b*) can be easily corrected by the so-called magic finger scheme (Hoyer *et al.*, 1995 ▸), whose result is shown in Fig. 6 ▸(*c*) with the required specification indicated by dashed lines.

**Figure 7:**
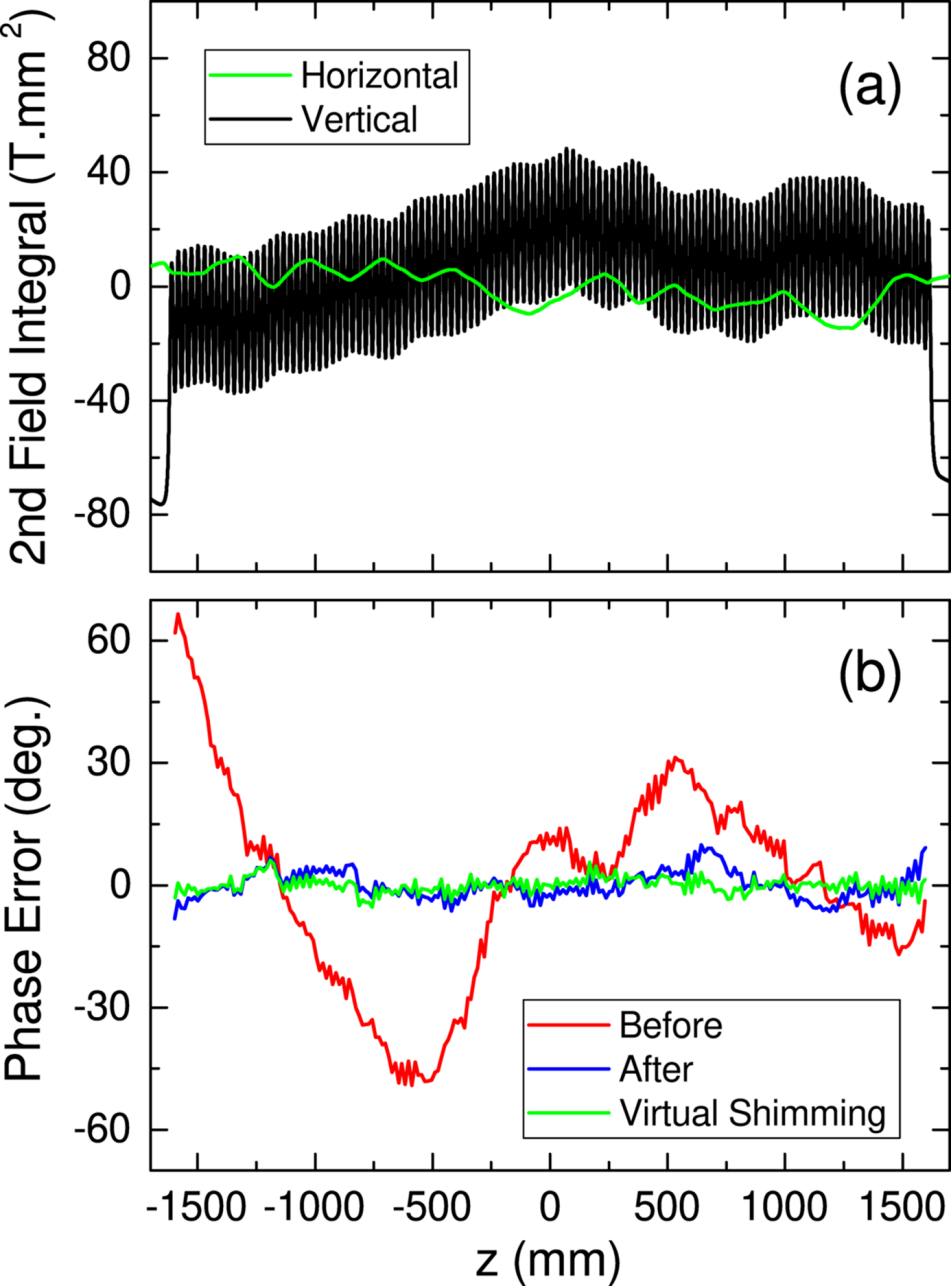
Magnetic quality of IVU-II28: (*a*) second field integrals just after assembly, and (*b*) phase errors before (red) and after (blue) correction; the green line in (*b*) shows the result of virtual shimming.

To examine the performance and quality of the assembled magnetic array, we measured the field distribution using a Hall probe sensor at a gap of 5.8 mm. The deflection parameter (*K* value) evaluated from the measured result is *K* = 3.27, which is consistent with *K* = 3.22 given by an analytical formula with an assumption of *B*_r_ = 1.37 T. The green and black lines in Fig. 7 ▸(*a*) show the second field integrals in the horizontal and vertical directions, respectively, evaluated from the measured data. Recalling that no corrections are made except for the module sorting, it is reasonable to say that the sorting works fine to reduce the trajectory wander in this example. The red line in Fig. 7 ▸(*b*) shows the phase error evaluated from the same data, where we find a systematic error that can be easily corrected in two steps. First, the overall trend is corrected by adjusting the local gap values at the three different positions of the bellows shafts. Second, the thickness of the shim plate inserted at the bottom of each module is optimized to correct the local gap variation. To be more specific, a number of shim plates, with the initial thickness being evenly 0.1 mm, are replaced with thicker or thinner ones (with a resolution of 0.01 mm) to correct the systematic phase error. The resultant phase error is shown by the blue line in Fig. 7 ▸(*b*), with an RMS (root-mean-square) phase error of 3.5°, which is usually acceptable in most applications.

Note that the phase error correction [from the red to blue lines in Fig. 7(*b*)] has been carried out in a few days in this example because of a tight construction schedule. Further reduction of the phase error is definitely possible if we have a longer time for correction using shim plates with a better resolution. For example, Fig. 7 ▸(*b*) shows a numerical result of such ‘virtual shimming’ evaluated by an assumption that the thickness of the shim plate is optimized with a resolution of 0.005 mm, in which case RMS phase error is reduced to as low as 1.9°.

**Figure 8:**
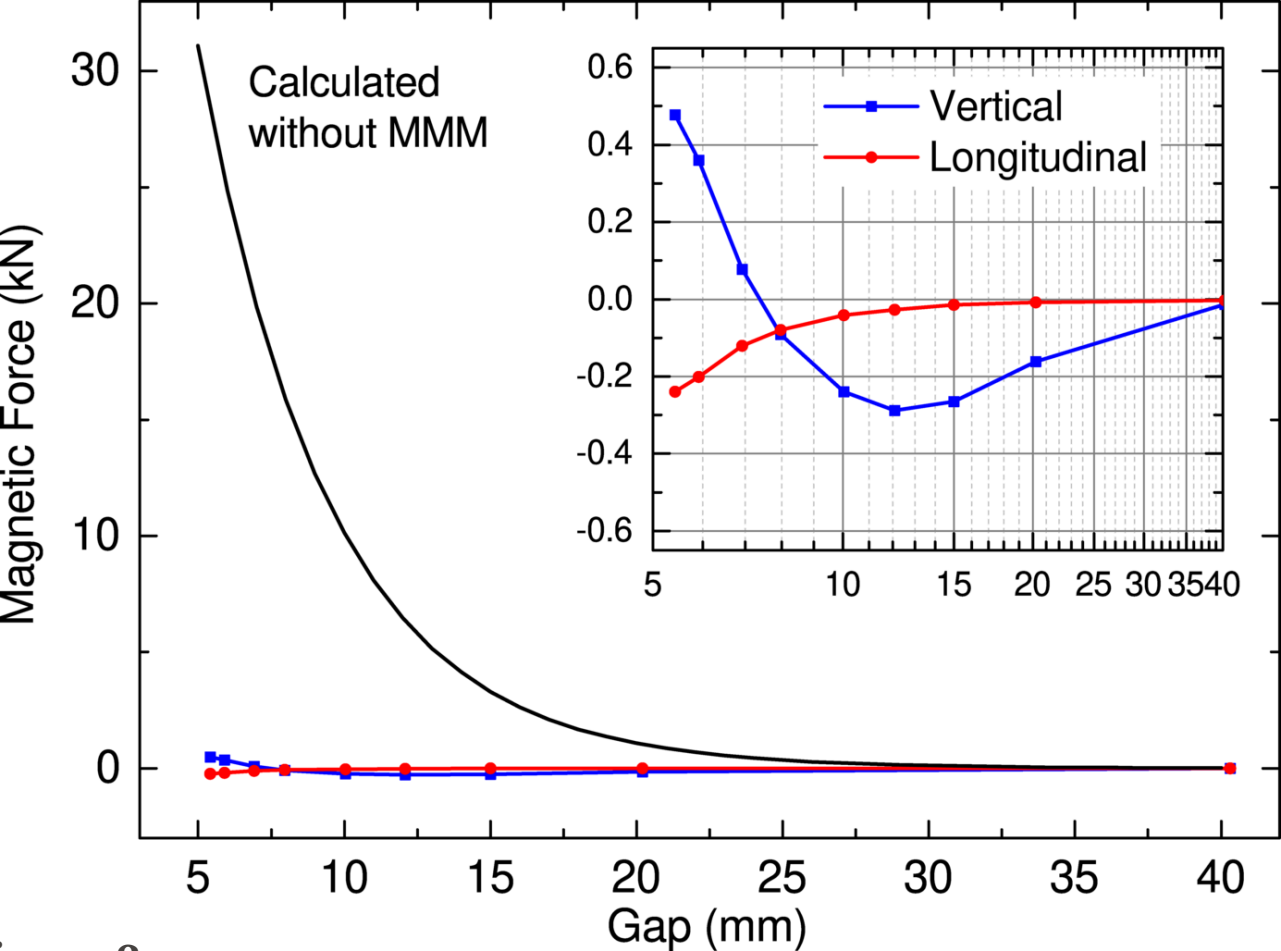
Results of the magnetic force measurement. The blue and red lines show the measured magnetic forces in the vertical (blue) and longitudinal (red) directions, while the black line shows the attractive force without the MMMs calculated with an analytical formula.

In addition to the magnetic performance and quality mentioned above, we have to check whether the mechanical load on the magnetic array is within an acceptable level. For this purpose we built a test bench to measure the magnetic force acting on the magnetic array as a function of the gap. The result is plotted in Fig. 8 ▸, where the blue line and squares show the magnetic force along the vertical direction with a positive/negative value indicating an attractive/repulsive force. For reference, the attractive force calculated using analytical formula is shown by the black line. Obviously, the mechanical load is significantly (nearly two orders of magnitude) reduced by the MMMs.

It is worth noting that the vertical residual force (blue) is not monotonic and the sign is flipped around the gap of 7 mm, which means that the repulsive force has a component whose gap dependence is not exactly the same as that of the attractive force. This is probably attributable to the extra (magnetically blank) space at the junctions of the magnet modules, which causes a longer-period component. From a practical point of view, however, this effect can be ignored because the residual force is smaller than the weight of the magnetic array (∼600 N).

Besides the attractive force, we have to take care of the longitudinal force, which is not usually found in conventional IVUs. As reported before (Kinjo *et al.*, 2017 ▸), a misalignment of MMMs, *i.e.* a longitudinal shift between the top and bottom MMMs, gives rise to an unexpectedly large longitudinal force, in particular when the misalignment is systematic. As shown by the red line and circles in Fig. 8 ▸, the maximum longitudinal force in this example is around 200 N, which is so small that no special care is needed.

## 5. Summary

We have presented the development of IVU-II, a cost-effective IVU with a high performance, which is based on three key technologies for overcoming the technical issues in conventional IVUs. The magnetic performance and quality achieved in IVU-II28 demonstrated the effectiveness of these technologies. Note that IVU-IIs with λ_u_ = 22, 24 and 28 mm are under construction to be ready for the SPring-8-II project, and most of the IVUs currently in operation in SPring-8 will be replaced with one of the IVU-IIs according to the required spectral range.
